# Making Reflective Practice by Interns Visible Through Digital Storytelling

**DOI:** 10.1007/s11606-026-10217-9

**Published:** 2026-02-25

**Authors:** Rebecca M. Forrest, Thomas C. Iden, Cherie Edwards, Claire Kimberly, Kenneth Warren Foster, Bennett Lee, Stephanie Call

**Affiliations:** 1 Department of Internal Medicine and program Director of the Internal Medicine Residency Program, Virginia Commonwealth University/VCU Health System, Richmond, VA USA; 2 University of Chicago, Chicago, IL USA; 3 Valley Health System, Winchester, VA USA

## Abstract

**Background:**

Facilitated reflection-based activities may support the development of reflective practice in medical trainees, an important activity in the process of professional identity formation. Digital storytelling is a multimedia reflection-based activity that expands upon traditional modalities and follows universal design for learning. There is a paucity of literature describing use of digital stories in graduate medical education trainees.

**Objective:**

We explored themes in digital stories by interns reflecting on experiences that impacted and shaped them as physicians.

**Design:**

We performed a retrospective thematic analysis of digital stories created by Internal Medicine interns to describe themes of transformative learning experiences.

**Participants:**

All interns within a single residency program and at large, university-based health system participated in a reflection curriculum between July 2015 and June 2019. We considered 161 digital stories created during this time period for thematic analysis until we achieved saturation of themes.

**Approach:**

To foster understanding of the rich, complex data within digital stories we used an inductive approach to construct themes.

**Key Results:**

We identified three themes from thirteen focused codes. Digital stories revealed interns use this modality to deeply describe experiences in which they (1) realized an aspect of their professional identity, (2) further understood or engaged with patients, (3) or recognized the challenges of the physician role.

**Conclusions:**

To our knowledge, this is the first thematic analysis of digital stories by graduate medical education trainees. We observed that digital storytelling gave interns an opportunity to reflect upon experiences informing their professional identity and patient stories. This activity allowed interns to share and understand challenges they faced. These themes align with the reflection prompt, indicating we met the curricular objectives, as well as key domains in professional identify formation. Through the rich themes identified, we demonstrate this unique technology-based modality effectively fosters deep, intentional, explicit reflection.

**Graphical Abstract:**

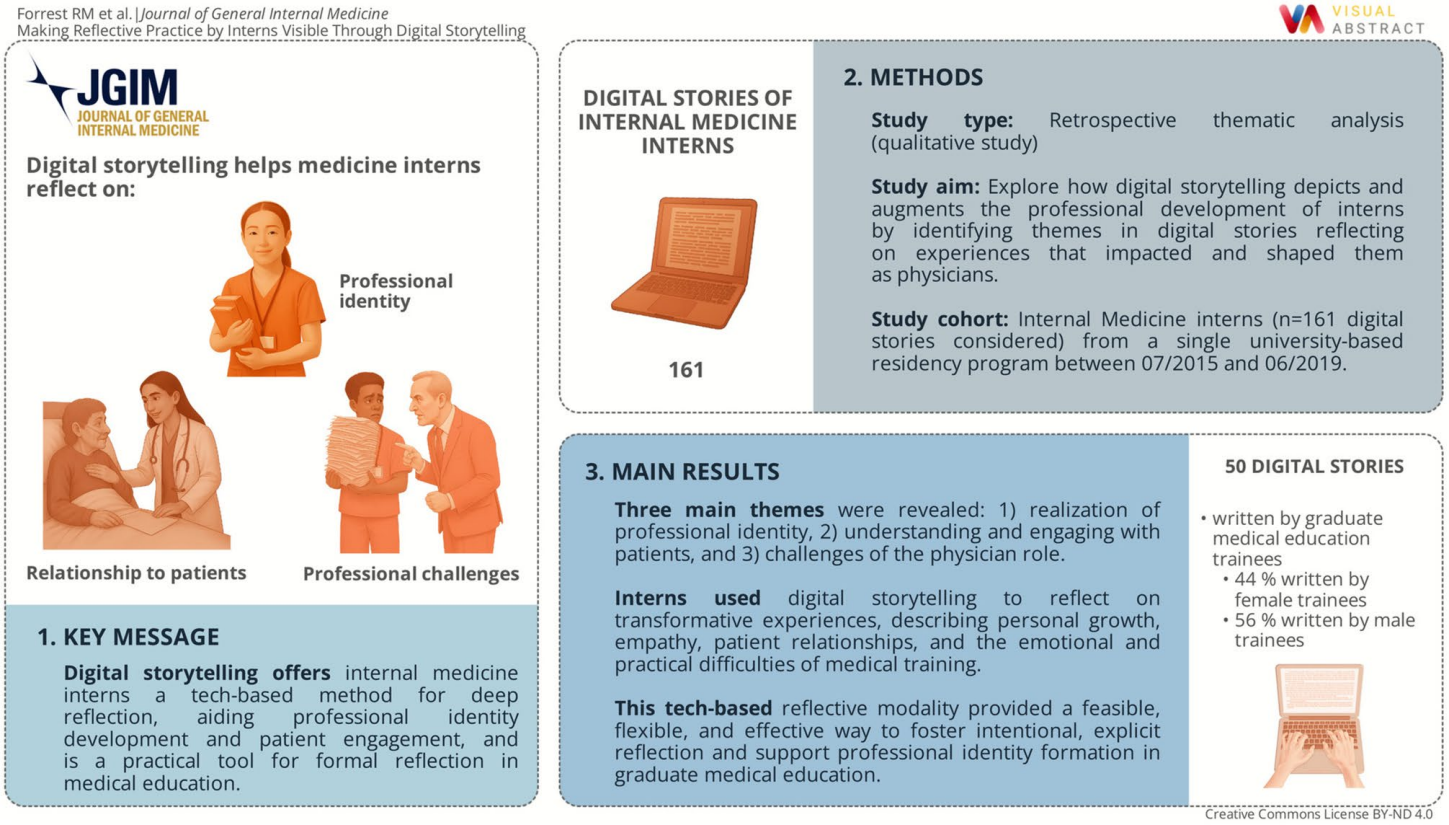

**Supplementary Information:**

The online version contains supplementary material available at 10.1007/s11606-026-10217-9.

## INTRODUCTION

Reflection is a cornerstone of physician training and practice. Regular reflection contributes to professional identify formation (PIF), competence, and acquisition of knowledge and wisdom.^1–4^ PIF is the developmental process through which learners internalize the values, behaviors, and sense of belonging essential to the physician role. It is widely recognized as a central construct in medical education, shaping how trainees integrate personal meaning with professional norms. Narrative medicine and storytelling with reflection are activities that enhance learning and may ultimately improve patient care.^5,6^ These practices foster the development of desirable characteristics of physicians (empathy, professionalism, and trustworthiness) and adaptive expertise and knowledge acquisition in a low stake environment.^7,8^ Facilitated and shared reflection-based educational activities in medical education may be important for guiding learners in developing reflective skills and advancing PIF.^9^

Evidence strongly supports narrative medicine interventions—including reflective writing, storytelling, and facilitated group discussions—as effective strategies for fostering self-exploration, empathy, and awareness of professional identity among medical trainees. Huang et al. found that narrative medicine facilitated self-exploration, reflection, and heightened awareness of professional identity, which in turn supported professionalism learning in clinical settings.^10^ Similarly, a realist synthesis by Huang et al. demonstrated that narrative medicine activities enhanced relational competence, empathy, and reflective practice, all of which are central to holistic professional identity development.^11^ Longitudinal approaches, such as narrative portfolios and meta-reflection exercises, provide structured opportunities for learners to examine evolving identities and gain perspective on professional growth.^12,13^ Faculty and peer narratives further reinforce socialization processes, promoting humanistic values and resilience.^14,15^ Systematic reviews confirm these findings, identifying reflective writing and narrative reflection as dominant modes of PIF intervention in medical education.^3,16^

Digital storytelling is a less-broadly used signature pedagogy that promotes critical reflection and emotional engagement in a safe space for collaboration and learning.^17^ Participants write and then record short, first-person narratives. They then combine the two to four-minute audio recordings with images, music, and other sound effects. These media utilize accessible, and often open, technologies to make reflection, via storytelling, visible and tangible.^18^ Resulting videos can be shared with peers for further review and interpretation of meaning. This unique modality offers a learning opportunity that supports both the individual and social-contextual approach to professional identify formation.^16^ The appropriate use of these tools can foster creativity by allowing authors to craft agency around visual metaphors (photography, illustrations, videos, etc.) and auditory elements (music, sound effects, vocal inflection, etc.) that may amplify or identify new points of learning. The use of multimedia with reflection aligns with Universal Design for Learning Guidelines, engaging learners through utilizing different types of media and allowing them to represent their perspectives authentically.^19^ This form of reflective practice also allows learners to meet the highest level of learning objectives within Bloom’s taxonomy through developing and creating knowledge.^20^

Sandars et al. describe a framework for implementing digital storytelling with medical students to foster reflective practice including but not limited to the following steps: decide on a topic, write the story, collect and select multimedia, create the story, present the story, encourage reflection at all stages.^21^ Other tips shared by Sandars et al. are to have achievable goals, provide technical support, have an evaluation for the process, and to consider including the practice in other elements of an existing curriculum.^21^ Educators may use digital stories for a stand-alone reflection assignment or they may be incorporated into other medical education curricula such as medical humanities, quality and safety, or advocacy curricula. Digital stories may be created and shared during small group, in person sessions or created independently with asynchronous sharing and dialogue. In a systematic review, 16 articles described using digital storytelling in health profession education (HPE).^22^ This review describes positive impacts on knowledge and understanding, including the ability to learn by listening to different perspectives and through creation of the story.^22^ The studies included the use of digital storytelling in graduate nursing (50%) and medical (6.3%) education, but there were no studies using digital storytelling with post-graduate physicians in training.^22^

### Objective

In this study, we explored how digital storytelling could depict and augment the professional development of interns by identifying themes in digital stories reflecting on experiences that impacted and shaped them as a physician.

## METHODS

### Study Design

We conducted a retrospective thematic analysis study which reviewed projects that resulted from an assignment that was a part of our reflection curriculum for interns in the Virginia Commonwealth University Health System (VCUHS) Internal Medicine residency program in Richmond, Virginia, United States of America.^23^ To foster understanding of the rich, complex data within digital stories we used an inductive approach to construct themes. Inductive thematic analysis facilitates understanding of “experiences, thoughts or behaviors across a data set” by revealing and analyzing themes to produce results.^23^ In inductive thematic analysis the process is data driven so themes are strongly linked to the data rather than being manipulated to neatly map back to the study’s conceptual framework or research questions.^24^ The Virginia Commonwealth University Institutional Review Board gave the study an exempt determination (study ID HM20014940).

### Setting and Participants

Between July 2015 and June 2019, Internal Medicine interns in the residency program participated in a reflection curriculum which includes the creation of a digital story. This timeframe reflects a period of stability in the residency program curriculum and leadership and preceded the COVID-19 pandemic. The reflection curriculum is comprised of four 1–2-h sessions facilitated by one of three authors who are faculty members in program leadership (Call, Forrest, and Iden). Four to five interns attend one session per week over the course of a four-week ambulatory rotation. Session content includes an introduction to reflective practice; active reflection via discussion, writing, and reading narrative medicine (session 1); an introduction to digital stories with a review and reflection on examples of previously created stories (session 2); an overview of using the technology to create a digital story and support in the first stages of creating their own story (session 3); and sharing and reflecting upon their own and one another’s digital stories (session 4). The digital story process follows the steps described by Sandars et al. ^16^ Prior to session 1, faculty send curriculum objectives to interns within an introductory email. During session 2, faculty share a rubric to guide best practice for creating digital stories. Between session 2 and session 3, interns receive a writing prompt and are instructed to return to session 4 with a 350–400-word reflection that is no more than three minutes when read aloud. Interns are asked to write about a patient, experience, or interaction that has changed their perspective of themselves, their role as a physician, or medicine. Between sessions 3 and 4, the interns use the technology to work independently on finalizing their digital stories. During session 4, interns share and reflect on digital stories within small groups of peers and a faculty facilitator. Faculty facilitators use the rubric shared during session 2 to guide group reflection and formative feedback. To promote authenticity and vulnerability, the program foregoes any formal, documented evaluation of the digital stories and requires participation purely as a learning activity. Curriculum materials, including learning goals and objectives and the rubric, are available in the supplementary materials. All digital stories were created and stored in a secure web-based platform called WeVideo.^25^ Only the intern whom created the digital story and the three faculty facilitators had access to the videos.

### Data Analysis

**Figure. 1:**
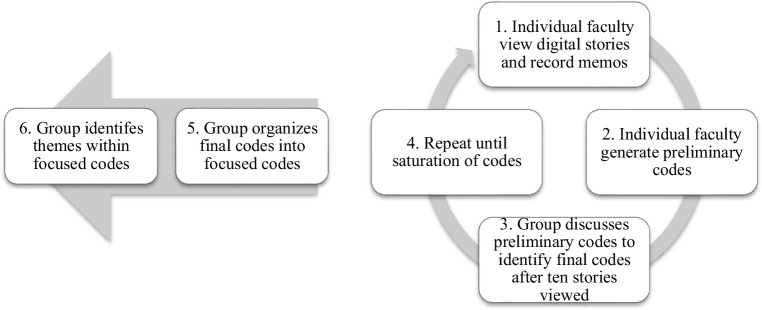
Methods flow chart**.**

We considered 161 digital stories created during this time period for thematic analysis until we achieved saturation of themes. We conducted an inductive thematic analysis (Fig. 1) to identify digital story themes using methodology described by Saldaña.^23^ To ensure credibility and confirmability, we did not limit themes to a preconceived list or framework and allowed all potential themes to arise. Prior to data analysis, we created unique identifiers by sorting the digital stories by date of creation and numbered each starting with “1” for the oldest and counting sequentially from oldest to newest. Three faculty (Call, Forrest, and Iden) viewed digital stories using a random-number generator to select the identifier and performed attribute coding of evident demographics once the story was viewed to ensure maximum heterogeneity among the digital stories reviewed. Attribute codes included the date of creation and gender of the resident author.

To generate themes inductively, each of the three faculty separately recorded analytic memos as described by Saldaña about observed content within the stories, any related observations (such as memories of interactions with participants during or outside of reflection curriculum, etc.) and reflections (reactions, perceptions, ideas that arose during viewing).^25^ Based upon the memos, faculty individually assigned preliminary concept codes, defined as”tentative ideas for codes, topics, and noticeable patterns or theme” using simultaneous coding “which applies two or more codes within a single datum.^23^” After independently viewing and assigning preliminary codes, the faculty discussed the preliminary codes to collectively identify final codes via consensus. To recognize when they reached saturation of codes, faculty discussed preliminary codes and assigned final codes via consensus in sets of ten videos. They stopped reviewing stories when they noted saturation of final codes. The faculty then collectively categorized final codes into focused codes which categorized “coded data as an initial analytic strategy” and lastly, reached consensus upon themes within the focused codes.^23^ To enhance credibility and trustworthiness, two authors (Warren Foster and Lee) familiar with digital stories and using similar curricula in undergraduate medical education and faculty development at our institution reviewed the analysis process and interpretations of the raw data.

### Ethical and Quality Issues

We took several measures to ensure trustworthiness, as defined by Nowell et al.^24^ We protected participant confidentiality by having only faculty who already view digital stories as part of the reflection curriculum participate in coding and we did not use any participant identifiers. This created multiple roles for the project facilitators that posed potential for bias in interpretation as the faculty viewers were the residency program director and two associate program directors who developed and run the reflection curriculum. To maintain authenticity and account for subjective influences, we recorded observations and reflections and had reflexive discussion throughout data analysis. We avoided potential bias that could arise from sequential sampling of digital stories within similar cohorts by using random sampling to promote heterogeneity of the digital stories viewed. All three faculty viewed all stories and participated in triangulation- discussing preliminary codes and assigning final codes together—to avoid impact of bias based upon personal experiences and conflict of interest between viewer and story writer.

## RESULTS

### Participant Characteristics

We identified 161 digital stories available to review. We reached saturation of themes after viewing a total of 50. We viewed 50 digital stories created in the following time periods: 18% from academic year 2015 (*n* = 9), 20% from academic year 2016 (*n* = 10), 34% from academic year 2017 (*n* = 17), 28% from academic year 2018 (*n* = 14) and 20% from July through September (*n* = 10), 32% from October through December (*n* = 16), 22% from January through March (*n* = 11), and 26% April through June (*n* = 13). Of those 50 digital stories, female interns created 44% (*n* = 22) and male interns created 56% (*n* = 28).

### Digital Story Themes

**Figure. 2:**
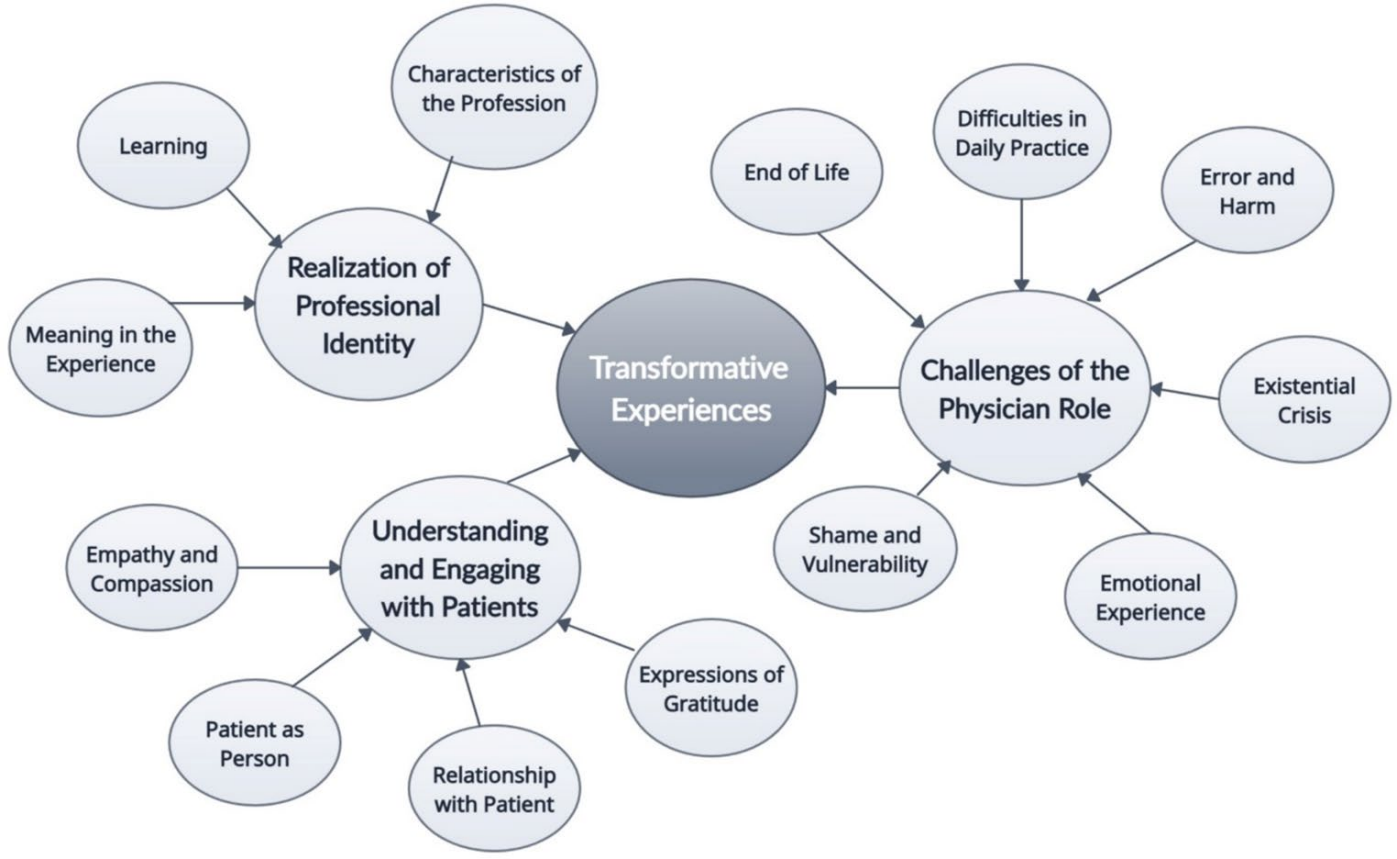
Transformative learning experience themes generated from focused codes**.**

In each story, we identified typically identified 3–5 final concept codes that were categorized into thirteen focused codes (Table 1). Within the thirteen focused codes, we identified three themes: (1) realization of professional identity; (2) understanding and engaging with patients; and (3) challenges of the physician role (Fig. 2). The themes are defined and supported by representative quotes in Table 2.

**Table 1:** Focused and initial codes by themes

**Realization of Professional Identity**		
Focused Codes	Final Codes	
Characteristics of the Profession	Enormity of role of physician Weight of responsibility Burden of responsibility Ownership Recognition of role as a physician Privilege Trust in physicians Pressure of responsibility Nobility of service Advocacy Serving patients Rewards of practice of medicine	
Learning	Learning from patient Listening Patient as teacher Lessons learned	
Meaning in the Experience	Appreciation Finding meaning Wonder/awe Inspiration Doctor as patient Sanctity of life Purpose Beauty Joy Hope Faith	
**Understanding and Engaging with Patients**		
Focused Codes	Final Codes	
Empathy and Compassion	Patient perspective Empathy Valuing needs and priorities Understanding Asking why Meeting needs of patient Self-centered physician Patient’s insight into own illness Burden of illness/disease Challenges and vulnerability of patient Loneliness of disease Hardening Lack of compassion Mindfulness	
Patient as Person	Humanity/humanism Lack of bias/judgment Partnering with patient Shared decision Engaging in patient care Depersonalization Autonomy Patient choice Social determinants of health Listening and hearing Assumptions	
Relationship with Patient	Connection with patient or family Relationship Therapeutic relationship Time spent with patients is therapeutic	
Expression of Gratitude	Thanking to patients or families Gratitude for the experience Generosity of patients Gratitude for the opportunity	
**Challenges of the Physician Role**		
Focused Codes	Final Codes	
End of Life	Patient fear Decision to change goals of care Counseling patients and families Delivering bad news Grief and loss Pronouncing patient deaths	
Difficulties in Daily Practice	Intern experience Comradery Consumption of self by residency Stress Demands Overwhelm of repetition Burden of routine Daily routine Efficiency Naivety Workload Shared experience with colleagues Challenges of practice of medicine Residence fatigue Lack of time Limitations of medicine Realistic expectations Roller coaster of critical illness Illness trajectory Complexities of illness	
Error/Harm	Complication Mistake/error Risk of harm	
Existential Crises	Uncertainty Questioning Moral conflict Futility Failing to fix Cruelty of fate Fragility of life	
Emotional Experience	Emotional burden/response on individual Anger Grief Frustration Helplessness Joy Accordion of hope (ups and downs) Acceptance Powerlessness, desperation Fear of physicians Impotency	
Shame/Vulnerability	Apology Imposter syndrome Shame Vulnerability Guilt Self-doubt Sense of failure Regret

**Table 2:** Representative Quotes from Digital Story Themes

**Realization of Professional Identity**	
Definition	Representative Quotes
Discovery of aspects of the physician’s role and the processes of learning and finding meaning in experiences	*1. “I was less than a week into my internship. I had been awake for almost 24 h. My mind was refractory to caffeine and I couldn’t wrap my head around your metabolic problems lab abnormalities which had to be corrected by morning. Every time my pager went off and I saw your name, I wanted to cry. I was paralyzed by my fear. Not for you- I couldn’t hurt you as who you once were was essentially gone. It was fear of harming your mother, father, grandparents, siblings, the people who love you most in the world and who are about to lose you forever. I imagine their only solace was knowing that you were going to better lives of others. And then there were the others…”*
	*2. “As I turned to walk out of the room he quietly said, I just don’t want you to think I’m dumb. This caught me off guard so I turned around and asked him what he meant. He then started to speak to me at length about his accomplishments. He spoke about how he built several famous buildings throughout the world and he worked throughout his adult life to be a great architect. He said that he wanted his young doctor to know that he had not always been this way. I didn’t quite know what to say. As a physician I have seen the damage that dementia can do. As a grandson, I know how hard it is to watch someone slowly slip away however, all this time I have never stopped to think about how the individual suffering from dementia as their memories slowly faded.”*
	*3. “One day, the patient’s mother said to me, ‘Doctor you’re here all the time,” and I felt somewhat embarrassed, because truly they were the ones who were there all the time. They had become my inspiration. They- these people who tirelessly care for their families and loved ones- they who are fighting for their lives, and they who support them and doing so are my inspiration.”*
**Understanding and Engaging with Patients**	
Definition	Representative Quotes
Expressions of empathy and compassion, gratitude for patients and recognition of humanism in medicine	*4. “We opened up a textbook and found the section entitled chest pain, but you did not fit a textbook. Nor was the chest discomfort the root of your real pain. We did not immediately hear your real concerns. Your tears were truly concerned for your 18-year-old daughter home alone, taking care of your one-year-old granddaughter, homeless, and alone in a motel room with no money to even pay for another night and no friends or family to take care of their needs. Your chest pain became secondary to the needs at home.”*
	*5. “I thought through what I would say to her this visit, how I would again broach the subject of her smoking, and recommending very strongly that she needed to get this chest CT for screening. It was what was best for her. She wasn’t gonna blow me off this time… As I walked into the room, she looked up at me and before I could get any words out, she said, ‘Doctor I wanted to tell you something. I just decided to quit smoking permanently.’ This time, I quickly sat down as I pulled up my chair across from her. I stared into her face, and she told me about her sister, who had recently been hospitalized, and diagnosed with cancer.”*
	*6. “Around the time I answered their 500th question I realized that I had started enjoying explaining things to Mr. S and his wife. It had become an exciting challenge to myself to translate all the medical work up and treatments we were doing into terms they could understand. I also came to appreciate the real reason they were asking so many questions. They were afraid of what was going on scared there was something wrong with Mr. S.—that his health was truly at risk.”*
**Challenges of the Physician Role**	
Definition	Representative Quotes
Descriptions of emotional, mental, and physical trials as physicians	*7. “After 30 min for seven patients in the morning, the rest of the day patients are virtual. They feel more like a concept of illness, a somewhat abstracted problem more than flesh and blood. As I become a technician and not writing bureaucrat and my patient becomes a series of ailments that I must come to terms with and manage. It can be alienating as the institutional apparatus creates distance between my personhood as a doctor and the person I should be helping.”*
	*8. “She wanted to go home. But Miss Laura did not have a social support system and her long-term facility options were limited by a lack of insurance. She lives in a housing development that was burdened by violence and stricken by poverty. She did not have reliable access to food or heat. In the hospital, she would have food and more. But Miss Laura’s disease was so far advanced she did not want to eat. In the hospital, she was surrounded by people who could monitor her – but they were strangers. In reality, modern medicine is dangerous and misleading…I couldn’t help but think how this isn’t much safer than Miss Laura’s home.”*
	*9. “I didn’t have the answer to some questions, but I do remember her face when you mouthed the words one day, ‘Just let me go. Just let me die.’ You pointed at the ventilator, ‘just take them off.’ That was an emotional time for me. I had been caring for you since you first had shortness of breath, since before you even needed a vent. It was crushing for me to see someone that was not delirious make a decision like that.”*
	*10. “‘Thank you for your service.’ What service? I wasn’t helping people. I was ending lives. Those five words go through my head as I stood at bedside calling yet another death. I heard those words again as I caught my patient’s daughter in my arms after she collapsed from the shock of watching her mother died. I heard them again as I comforted her, reassuring her that she had done right by her mother, and let her pass in peace with dignity without pain.”*

### Realization of Professional Identity

The theme “Realization of Professional Identity” includes descriptions of the characteristics of the profession, examples of learning and finding meaning in the different experiences. Coders noted, “appreciation, enormity of what we do,” as well as the “weight of responsibility,” and “burden…of doctoring’ in representative memos. For example, one intern described her experience of managing a critically ill patient through the night so that he would be a viable organ donor when removed from life support the following day. The intern recounted, “my mind was refractory to caffeine and I couldn’t wrap my head around your metabolic problems lab abnormalities which had to be corrected by morning*”* (Table 2, Quote 1). Often, the patients were described as teachers, with memos describing, “patient helping doctor” and “patients as educators.’ Interns derived meaning from experiences, with memos recording examples of “finding a purpose” and “wonder/awe” and “appreciation.” In one story demonstrating wonder and awe, an intern described his admiration for the dedication of patients’ families to caring for and supporting loved ones through their illnesses. The intern stated, “they- these people who tirelessly care for their families and loved ones- they who are fighting for their lives, and they who support them and doing so are my inspiration” (Table 2, Quote 3).

### Understanding and Engaging with Patients

Stories describing expressions of empathy, compassion, gratitude and highlighting humanism in the interns’ experiences generate the theme “Understanding and Engaging with Patients.” Memos from this theme include “meeting patients where they are” and “socioeconomic determinants of health.” In a story reflecting on meeting the needs of an individual patient, an intern described a patient wanting to leave the hospital, despite having a very grave illness, to be able to travel home and receive care where family could support him. Coders recorded many examples of a “patient as person,” the “impact of bias,” and medicine “undermining humanity of patients.” In one example, an intern told the story of a patient with a life-threatening condition leaving against medical advice due to her primary concern for her children alone at home, realizing the patient’s “tears were truly concerned for your 18-year-old daughter home alone, taking care of your one-year-old granddaughter, homeless, and alone in a motel room” (Table 2, Quote 4).

Digital stories described connections and relationships with patients and their families as well as the therapeutic value of spending time with patients. Coder memos included, “listening to patients,” the “role of family,” and “importance of connection.” In an instance of listening to a patient whom previously declined cancer screening one intern describes, “I stared into her face, and she told me about her sister, who had recently been hospitalized, and diagnosed with cancer” (Table 2, Quote 5). Interns used digital stories to explicitly say “thank you” to patients and their families, and recognize the generosity of patients. One intern recounted becoming grateful for the challenge of answering a multitude of daily questions and the opportunity to spend time with and comfort the patient. The intern ultimately appreciated “the real reason they were asking so many questions” was that “they were afraid of what was going on scared there was something wrong” (Table 2, Quote 6).

### Challenges of the Physician Role

Stories sharing the difficulties of daily practice as an intern, experiences with medical error and patient harm, end of life care, shame, vulnerability, deep emotions, and existential crises culminated in the theme of “Challenges of the Physician Role.” Coders recorded descriptions of “the dehumanization and depersonalization driven by workload,” as well as “overwhelm of repetition” and “impotency and helplessness.” One story described the burden of tasks that took physicians away from the bedside, lamenting that it “can be alienating as the institutional apparatus creates distance between my personhood as a doctor and the person I should be helping” (Table 2, Quote 7). Other stories encapsulated facing uncertainty, moral conflict, failure to cure, and the fragility of life such as concerns for patient's safety within the health care system due to the risk of medical error, nosocomial infections, etc. in contrast to the dilemma of being unable to discharge the patient home safely. One intern narrated, “in the hospital, she was surrounded by people who could monitor her – but they were strangers [and] modern medicine is dangerous and misleading” (Table 2, Quote 8).

Many digital stories discussed the experience of delivering bad news, establishing goals of care, and patients’ the end-of-life experiences. Memos describe “suddenness of death and grief,” and physician role in the end of life.” An intern’s reflections on the experience of caring for a patient who decided himself to withdraw life support and be allowed to die, describing, “I do remember her face when you mouthed the words one day, ‘Just let me go. Just let me die.’” (Table 2, Quote 9). Other stories recounted mistakes, specific medical errors, complications, and the risk of harm to patients. Interns also recognized one’s own emotional responses to experiences such as joy, hope, grief, anger, frustration, desperation, helplessness, acceptance, and fear. Digital stories often recounted experiences that elicited shame and vulnerability with descriptions of apologies, imposter syndrome, unworthiness, guilt, self-doubt, having a sense of failure, and regret. In one story, an intern described being thanked for his hard work and feeling unworthy of that gratitude because “I wasn’t helping people. I was ending lives” when multiple patients were dying or had died on his team in the intensive care unit that day (Table 2, Quote 10).

## DISCUSSION

We identified three major themes in this study of an Internal Medicine intern digital story reflection assignment about an impactful experience in internship. These themes align with the intended assignment and prompt to reflect on professional transformation, indicating that this unique reflection activity is an effective modality to achieve the desired curricular outcomes. While digital storytelling has been described in undergraduate medical education, to our knowledge this is the first thematic analysis of digital stories by graduate medical education trainees.

We found a broad array of themes similar to those previously described in evaluations of reflective exercises in medical education and in studies of reflection of professional identity formation curricula or interventions. Prior studies of reflective practice in medical students and trainees describe themes such as empathy for and consideration of the patient perspective (comparable to “Empathy and Compassion” and “Patient as Person”), facing uncertainty and finding confidence (comparable to “Existential Crises” and “Shame/Vulnerability), recognition of the professional role and identity (comparable to “Characteristics of the Profession”), emotions faced (comparable to “Emotional Experiences”), relationships and connections (comparable to “Relationship with Patient”), and expectations around and the impact of the workload (comparable to “Challenges in Daily Practice). As in descriptions of other reflection-exercises, medical trainees demonstrated self-awareness and a critical approach to training and work.^12,26,27^ These findings support the use of digital storytelling as additional, equally valuable mode of reflective practice in medical education. Offering digital storytelling with other modes of reflection adheres with principles of Universal Design for Learning which aim to engage and meet the needs of various styles, preferences, and abilities.^19^ Digital storytelling also provides an avenue of reflection that meets the demand to integrate technology-based pedagogies into medical education and training.

Intern digital stories also described how the author’s experience shaped one’s perspective or practice in the future. We found that digital stories provided interns an opportunity to realize aspects of their professional identity and to tell stories about their patients- often reminding them of their own uniqueness and that of the individuals for whom they care. This reflective exercise also provided an avenue for interns to share and seek to understand challenges they faced- fostering acceptance of and growth and learning from these experiences. These themes describe known facets of professional identity formation, supporting the use of digital storytelling as a valuable tool for graduate medical education trainees during a key phase of professional identify formation. In addition, the final sharing stage of the digital storytelling process fosters socialization and community of practice which are key components of professional identify formation. While not explicitly studied, reviewers noticed that the addition of digital media allowed for emphasis on and illumination of themes that otherwise may have been less apparent. For example, images of holding hands highlighted the theme of connection with patients and a change in music often signified a key aspect of the story or the transition to how the experience impacted the writer. These observations require confirmation through additional studies.

Our study is from a program that has an extensive longitudinal curriculum in resilience and reflection which may influence the results we found. In addition to the reflection curriculum, interns participate in monthly conferences in which they practice resilience skills and reflect on experiences. Additionally, this study is from a single specialty within a single institution, limiting the generalizability of our results to other specialties or programs with different structures or in different settings. Some themes we identified may arise from the specific patient population encountered in our institution. We analyzed older data to minimize the impact of other variables following academic year 2018–19 and acknowledge the older data limits generalizability. Our sample had a higher proportion of stories created in academic year 2017–18 and academic year 2018–19, likely due to the addition of preliminary medicine interns to the reflection curriculum that year.

As this is a qualitative study, the perspective and experience of individuals performing analysis may influence the evaluation. Specifically, the three faculty facilitators (Call, Forrest, and Iden) served dual roles by performing the data analysis. This introduces potential for those performing the analysis to have prior knowledge of the circumstances described in the stories and insight into the curriculum that impacts the interpretation of the data. Additionally, the power dynamic between interns and faculty may have influenced the authenticity of the stories. We took measures consistent with best practice in qualitative study design to minimize biased interpretation including reflexivity, using consensus to designate codes and themes, and external auditing by two authors who did not participate in the residency program curriculum or data analysis. Based upon the retrospective nature of this analysis, we were unable to perform member checking. We attempted to minimize the power dynamic by building trust and fostering vulnerability through low-stakes, introductory sessions in reflection and a stepwise process for writing and creating stories prior to the final sharing session. Additionally, we designed this is a learning activity with no formal assessment. Having faculty in program leadership facilitate the reflection sessions contributes to developing relationships among those faculty and interns and a positive program culture that encourages vulnerability and potentially mitigates the negative impact of the power dynamic on authenticity.

## CONCLUSION

We demonstrate the use of digital multimedia as a component of traditional written or discussion-based reflective activities. This unique approach fostered deep, intentional, explicit reflections with resultant themes found in other modes of reflection and professional identity development curricula. The addition of digital-media and use of a cloud-based video production tool follows current trends within medical education by incorporating technology into existing pedagogies and offering a form of reflection that meets a broader array of learning preferences. The unique nature of digital storytelling (making the reflection visible and more accessible for sharing) may supports both the individual and social-contextual approach to professional identify formation. Further work to explore the shared component and impact of this component is necessary.

We conclude that digital storytelling by graduate medical trainees is a feasible and successful tool to foster meaningful reflection that contributes to professional identify formation. Our findings support the use of this low-input, high-yield modality that overcomes the challenge of incorporating digital media and technology into medical education pedagogies. Likewise, the rich themes in interns’ digital stories reinforce the immense value of formal reflection-based activities for learners in medical education across the continuum. As such, we suggest that medical educators consider digital storytelling as one of multiple modes of feasible, flexible, and scalable strategies for reflection that overcome barriers of high intensity, time consuming and variable schedules to engaging these learners in formal reflection across the continuum of medical education.

## Supplementary Information

Below is the link to the electronic supplementary material.

ESM 1 (DOCX 21.8 KB)

## Declarations

### Prior Presentations

The authors have shared the Digital Story curriculum in national workshops and posters, but the findings of this study have not been presented or published previously.
