# Supplementary material for: Making Reflective Practice by Interns Visible Through Digital Storytelling
Source: J Gen Intern Med. 2026 Feb 25;41(7):1855–66. doi: 10.1007/s11606-026-10217-9 (PMC13176442; doi:10.1007/s11606-026-10217-9)
Supplement: Supplementary file 1 — (DOCX 21.8 KB) [file 11606_2026_10217_MOESM1_ESM.docx]

**Reflective Practitioner – Goals and Learning Objectives**

Goals: The goal of the reflective practice experience is to foster the development of the Internal Medicine trainee as a reflective learner. Through discussion about reflection and engaging in several modes of reflection, they will gain an understanding of the practice of reflection, an appreciation of the importance of reflection as a behavior for the developing and the practicing clinician, and experience several methods for reflecting that they may consider incorporating into their daily practice and learning activities.

Specific Learning objectives:

At the end of the reflective practice experience, the Internal Medicine resident will:

1. Be able to define “critical reflection”.
2. Be able to identify the value of critical reflection in learning and clinical practice.
3. Be able to list methods for engaging in and supporting regular critical reflection.
4. Be familiar with the literature in reflective activities for practicing physicians.
5. Be able to evaluate their own current practice in critical reflection.
6. Participate in multiple types of critical reflection activities, to include writing, discussion, critical incident reflection, and digital storytelling.
7. Demonstrate the capacity for critical reflection through a reflective activity that is shared with others.
8. Gain an appreciation for the role of critical reflection in daily practice and learning.
9. Set goals for ongoing practice of reflection.

**Digital Story Prompt**

Please prepare a story/reflection about a patient, experience, or interaction that has changed your perspective on you, on your role as a physician, or on medicine. The story should focus on you and your reflection and what you will take away from the experience. When written, the narrative should be about 1-1.5 pages double-space typed in length and around 2.5-3 minutes when read out loud.

**Reflective Practitioner Assessment**

Name of resident_____________________________ Evaluator/Date________________________

1. Participation/engagement – rubric

|  | Meeting expectations | Needs development | Unsatisfactory |
| --- | --- | --- | --- |
| Listening | Actively and respectfully listens to peers and facilitator | Sometimes displays lack of interest in comments of others | Consistently projects lack of interest in comments of others or disrespect for others |
| Demonstration of empathy | Acknowledges experiences of others and makes comments in support of others | Does not actively demonstrate support for others’ experiences, responses | Does now acknowledge experiences of others; makes comments that are dismissive or disrespectful with regards to peer experiences |
| Participation | Actively participates and engages at appropriate times | Sometimes participates; may “tune out” at other times or participate in a disruptive manner at times | Not engaged, does not participate or participates in a disruptive manner that interferes with session |
| Quality of contributions | Comments are relevant and reflect understanding or consideration of material, comments of others | Comments do not consistently reflect consideration of the material, comments do little to advance the conversation | Comments do not advance the conversation and reflect little understanding or attention to material |

1. Digital storytelling project – rubric

|  | Meeting expectations | Needs improvement | Unsatisfactory |
| --- | --- | --- | --- |
| Point of view – purpose | Establishes purpose, maintains focus | Few lapses in focus, but purpose fairly clear | Difficult to figure out purpose or focus of presentation |
| Voice – consistency | Voice quality is clear, consistently audible | Voice quality is clear and audible some of the time – not consistently | Voice quality needs more attention |
| Voice – pacing | Pace (rhythm and voice punctuation) fits the story | Occasionally speaks too fast or too slow – does not fit the story | No attention to rhythm and pace, does not fit the story |
| Soundtrack – emotion | Music stirs a rich emotional response that matches the story line well | Music is ok, does not add to story | Music is distracting or used inappropriately |
| Images | Images create a distinct atmosphere or tone that matches different parts of the story. Images communicate symbolism and/or metaphors. | Images match some parts of the story. There was an attempt to use images to create an atmosphere or tone, but needed more work. | Images are distracting or inappropriate. Images do not communicate symbolism. |
| Economy | Story is told with right amount of details – does not seem too short or long. | Story seems to need more editing, noticeably too long or short in sections | No attention to economy, needs significant editing or too short to convey message |
| Duration | Length of presentation 4 minutes or less | Length longer than 4 or shorter than 2 minutes | Presentation way outside of time expectations |
| Evidence of reflection | Story focuses on impact experience had on storyteller, personal. | Story is one that impacted storyteller but story is not told in personal manner or perspective | Story is not personal, objective, no evidence of reflection of impact on storyteller. |

Comments:
